# Increased QRS duration and dispersion are associated with mechanical dyssynchrony in patients with permanent right ventricular apical pacing

**DOI:** 10.15190/d.2021.7

**Published:** 2021-06-26

**Authors:** Elibet Chávez-González, Arian Nodarse-Concepción, Ionuț Donoiu, Fernando Rodríguez-González, Raimundo Carmona Puerta, Juan Miguel Cruz Elizundia, Gustavo Padrón Peña, Ailed Elena Rodríguez-Jiménez

**Affiliations:** ^1^Department of Electrophysiology, Cardiocentro Ernesto Che Guevara, Santa Clara, Villa Clara, Cuba; ^2^Department of Cardiology, University of Medicine and Pharmacy, Craiova, Romania; ^3^Cardiology Service, Camilo Cienfuegos Hospital, Sancti Spíritus, Cuba

**Keywords:** Cardiac pacing, echocardiography, left ventricular function, ventricular dyssynchrony.

## Abstract

Background: Permanent right ventricular apical pacing may have negative effects on ventricular function and contribute to development of heart failure. We aimed to assess intra- and interventricular mechanical dyssynchrony in patients with permanent right ventricular apical pacing, and to establish electrocardiographic markers of dyssynchrony. Methods: 84 patients (46:38 male:female) who required permanent pacing were studied. Pacing was done from right ventricular apex in all patients. We measured QRS duration and dispersion on standard 12-lead ECG. Intra- and interventricular mechanical dyssynchrony and left ventricular ejection fraction were assessed by transthoracic echocardiography. Patients were followed-up for 24 months. Results: Six months after implantation, QRS duration increased from 128.02 ms to 132.40 ms, p≤0.05. At 24 months, QRS dispersion increased from 43.26 ms to 46.13 ms, p≤0.05. Intra- and interventricular dyssynchrony increased and left ventricular ejection fraction decreased during follow-up. A QRS dispersion of 47 ms predicted left ventricular dysfunction and long-term electromechanical dyssynchrony with a sensitivity of 80% and a specificity of 76%. Conclusion: In patients with permanent right ventricular apical pacing there is an increased duration and dispersion of QRS related to dyssynchrony and decreased left ventricular ejection fraction. This study shows that QRS dispersion could be a better predictive variable than QRS duration for identifying left ventricular ejection fraction worsening in patients with permanent right ventricular apical pacing. The electrocardiogram is a simple tool for predicting systolic function worsening in these patients and can be used at the bedside for early diagnosis in the absence of clinical symptoms, allowing adjustments of medical treatment to prevent progression of heart failure and improve the patient's quality of life.

## INTRODUCTION

When cardiac pacing was discovered as a conduction disorder treatment, the greatest concern was the patient survival but without addressing other aspects^1,2^. Since 1925, Wiggers examined several epicardial pacing sites in dogs. A decreased left ventricular pressure during isovolumetric period contraction was associated with right ventricular apical pacing, whereas pacing close to His-Purkinje system did not produce deleterious hemodynamic effects. He postulated that pacing site exerted a considerable influence on left ventricular function and was probably the first that recognized His fascicle as an excellent place for permanent pacing^3,4^. In 1998, Van Oosterhout et al.^5^ characterized the morphological changes produced in the canine myocardium after pacing. The relationship between asynchrony and reduction of myocardial performance was postulated^6^. The greater percentage of right ventricular pacing accumulated over time is related with changes in left ventricular geometry and hemodynamic parameters. It has been widely evidenced that right ventricular apical pacing induces asymmetrical ventricular hypertrophy, ventricular dilatation, abnormal fiber arrangement, increase of cathecolamines and myocardial perfusion disturbances, among other harmful effects^4,7,8^.

Mode Selection Trial (MOST) study comparing dual chamber (DDD) and single chamber (VVI) pacing mode patients analyzed the relationship between the proportion of beats with ventricular pacing and hospitalization for heart failure or onset of atrial fibrillation^9^. It was found that, regardless of pacing mode, a high percentage of stimulated beats is a strong predictor of left ventricular dysfunction or atrial fibrillation development^9,10^. Heart failure secondary to right ventricular apical pacing has also been demonstrated^11,12^.

In patients with chronic heart failure associated with left bundle branch block (LBBB) and reduced left ventricular ejection fraction (LVEF), QRS duration and dispersion are increased. Also, in New York Heart Association (NYHA) Class II-IV heart failure patients with severe systolic dysfunction (left ventricular ejection fraction <35%), QRS dispersion was significantly higher in deceased patients than in survivors (54 ± 17 ms versus 46 ± 16 ms, p <0.02). Moreover, patients which died suddenly had significantly higher QRS dispersion than survivors (56 ± 13 ms versus 46 ± 16 ms, p <0.02). A QRS dispersion cut-off value of 46 ms separated patients with higher risk of death in the following three years (mortality 13% versus 32%, relative risk 3.85)^13^.

Based on the idea that the right ventricular apical pacing’s deleterious effect is associated with LBBB, generated by pacing itself, which causes electromechanical dyssynchrony, we aimed to determine the relationship between mechanical intra- and interventricular dyssynchrony, observed with echocardiography, and electrocardiographic changes in patients with permanent right ventricular apical pacing, regardless of pacing mode: dual chamber or single chamber.

## MATERIAL AND METHODS

### Study patients

Patients scheduled for permanent pacemaker implantation due to complete atrio-ventricular block, atrial fibrillation with low heart rate, carotid sinus hypersensitivity, hypertrophic cardiomyopathy, sinus node dysfunction were enrolled prospectively. Ventricular pacing was performed from the right ventricular apex, either VVI or DDD mode. Exclusion criteria were: left bundle brunch block, primary dilated cardiomyopathy, known ischemic heart disease or history of coronary revascularization, history of chronic alcoholism, prior chemotherapy and/or radiotherapy, exposure to cardiotoxic substances such as heavy metals, treatment with antiretroviral drugs, history of infiltrative diseases, patients who needed cardiac resynchronization therapy or implantable automatic defibrillator, corrective congenital cardiac surgeries or significant valvular heart diseases, patients who required antiarrhythmic in the last six months. The study included 84 patients.

**Table 1:** Distribution of patients according to pacing indication DDD: Dual chamber pacing, VVI: single chamber pacing.

Indication	Pacing Mode DDD (number / %)	Pacing Mode VVI (number / %)	Total (number / %)
Sinus dysfunction	20 / 23,81 %	6 / 7,14 %	26 / 30,95 %
Atrioventricular block	14 / 16,67 %	28 / 33,33 %	42 / 50,00 %
Carotid sinus hypersensitivity	2 / 2,38 %	0 / 0,00 %	2 / 2,38 %
Hypertrophy cardiomyopathy	2 / 2,38 %	0 / 0,00 %	2 / 2,38 %
Atrial fibrillation	0 / 0,00 %	12 / 14,29 %	12 / 14,29 %
Total	38 / 45,24 %	46 / 54,76 %	84 / 100,00 %

**Table 2:** Cardiac pacing and ECG variables during the follow-up

Statistics	Start of the study	6 months	12 months	24 months	t	p
**Frequency of pacing**						
Minimum	-	22	18	12		
Maximum	-	100	100	100		
Mean	-	85,15	83,81	85,04	0,13	0,895
Standard deviation	-	15,234	17,03	18,11		
						
**QRS duration**						
Minimum	50	100	100	100		
Maximum	130	180	180	180		
Mean	79,60	128,02	129,70	132,40	348,64	<0.000
Standard deviation	16,21	19,86	19,71	19,87		
						
**QRS dispersion**						
Minimum	28	30	30	30		
Maximum	44	77	80	80		
Mean	36,32	43,26	44,75	46,13	14,08	<0.000
Standard deviation	4,13	10,53	11,39	11,76		
						

### Data collection

Variables were recorded before pacing (with intrinsic cardiac rhythm), at six, twelve and twenty-four months after implant. From the standard 12-lead ECG QRS, maximum and minimum duration was manually measured by 2 independent observers, with a paper speed of 25 mm/s, amplitude of 10 mm/1 millivolts (mV). QRS was considered from the beginning of q or R wave up to J point at the beginning of the ST segment. QRS dispersion was calculated as difference between the QRS maximum and minimum value measured on the 12-lead ECG.

Standard transthoracic echocardiographic variables were recorded. Left ventricular ejection fraction was measured using biplane Simpson's method. All patients had left ventricular ejection fraction ≥ 50% at inclusion. For assessing left intraventricular dyssynchrony, electromechanical delay of septal-to-lateral wall was determined by means of tissue Doppler, locating the sample volume in the basal portion of the interventricular septum and lateral wall, in order to calculate the temporal difference between both, measuring from the beginning of the QRS complex until the peak systolic velocity. Echocardiographic demonstration of cardiac asynchrony was defined for values ≥ 60 ms^14-16^ in the four-chamber apical view, while interventricular dyssynchrony was considered according to the differences between left and right ventricle electromechanical delays (pulse-wave Doppler was used to measure the times from the beginning of QRS complex to the beginning of aortic or pulmonary ejection, as appropriate). Interventricular asynchrony (by means of the difference between these two calculated times) was defined as an aorto-pulmonary delay ≥ 40 ms^15,16^.

Pacing percentage was taken from pacemaker programmer.

### Statistical analysis

We used Statistical Package for Social Science (SPSS) version 21.0 for Windows. Frequency distribution tables with absolute and relative values were created. The mean was determined for the variables that required it with standard deviation as a measure of variability. For the comparison of means, the t-student statistic was used, and statistical significance was considered if p≤0.05. From the inferential point of view, the non-parametric Chi square test (χ^2^) was applied for association between variables with the same level of significance. Linear correlations were made (Spearman test). Discriminatory power of QRS duration and its dispersion were estimated using the receiver operator curve (ROC) using estimates and the 95% confidence interval (CI) and area under the curve was calculated.

The study protocol was approved by our institutional ethics committee and all patients gave informed consent.

## RESULTS

The age of the subjects included in this study was between 34 - 92 years with a mean of 68.25 ± 13.195 years. There were 46 males (54.76%) and 38 females (45.24%). Age distribution showed the highest number of patients had 70 or more years (46.43%), and between 60 and 69 years (28.57%). Pacing indication is summarized in Table 1. Atrioventricular block was the most frequent indication (50%), followed by sinus dysfunction with 30.95%.

**Figure 1:**
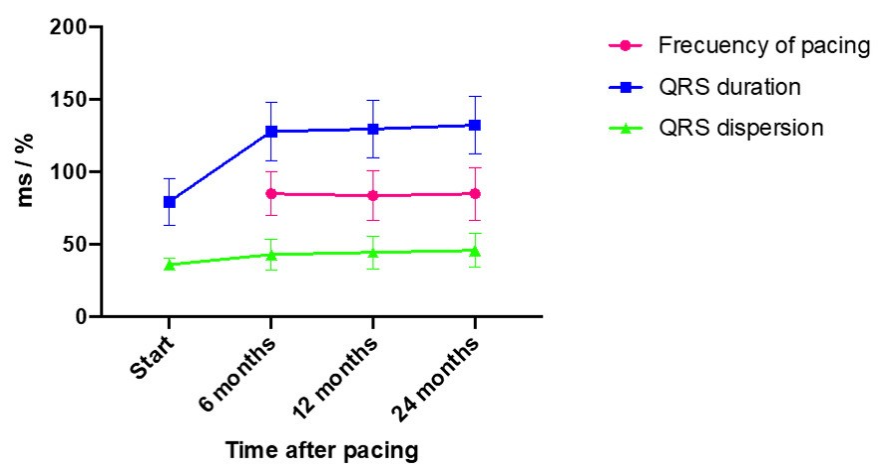
Means of cardiac pacing variables during follow-up

Frequency of pacing was greater than 83% in all patients. Table 2 and Figure 1 show the variables changes related to frequency of right ventricular apical pacing and QRS duration and its dispersion during the study. QRS duration was increased from 128.02 ms (six months after pacing) to 132.40 ms during two years of follow-up, p <0.000. QRS dispersion also increased from 43.26 ms (six months after pacemaker cardiac implant) to 46.13 ms after two years of follow-up, p <0.000.

**Figure 2:**
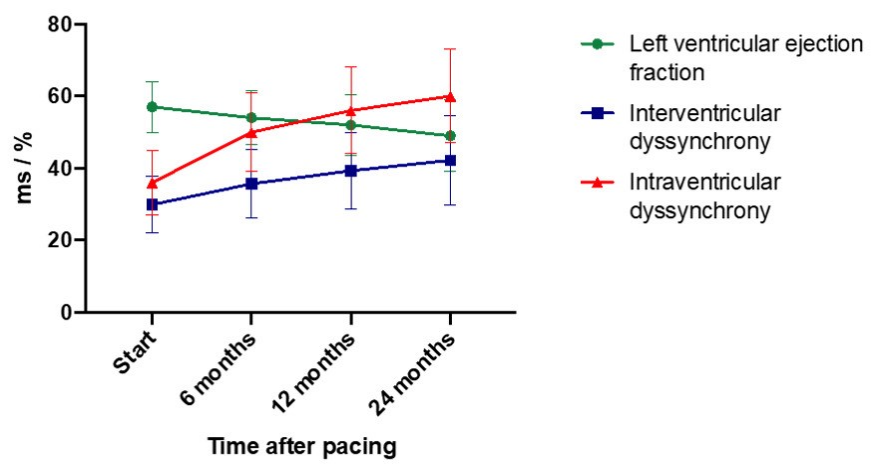
Evolution of echocardiographic parameters during follow-up

Figure 2 shows that interventricular dyssynchrony increased from 29.87 ms to 42.18 ms in the twenty-four months of follow-up, p≤0.05. Intraventricular dyssynchrony mean value increased from 35.82 ms to 60.29 ms, p≤0.05; left ventricular ejection fraction was reduced from 57.14% to 49.43%, p≤0.05.

QRS dispersion showed significant and positive correlations with interventricular dyssynchrony (p = 0.015) and intraventricular dyssynchrony (p = 0.001), as well as with pacing frequency (p = 0.003) (Table 3).

In addition, left ventricular ejection fraction significantly decreased, correlated with QRS dispersion (p = 0.016), Pearson coefficient=-0.606. Left ventricular ejection fraction also decreased when cardiac pacing percentage was high (p = 0.000), Pearson coefficient =-0.589.

**Figure 3:**
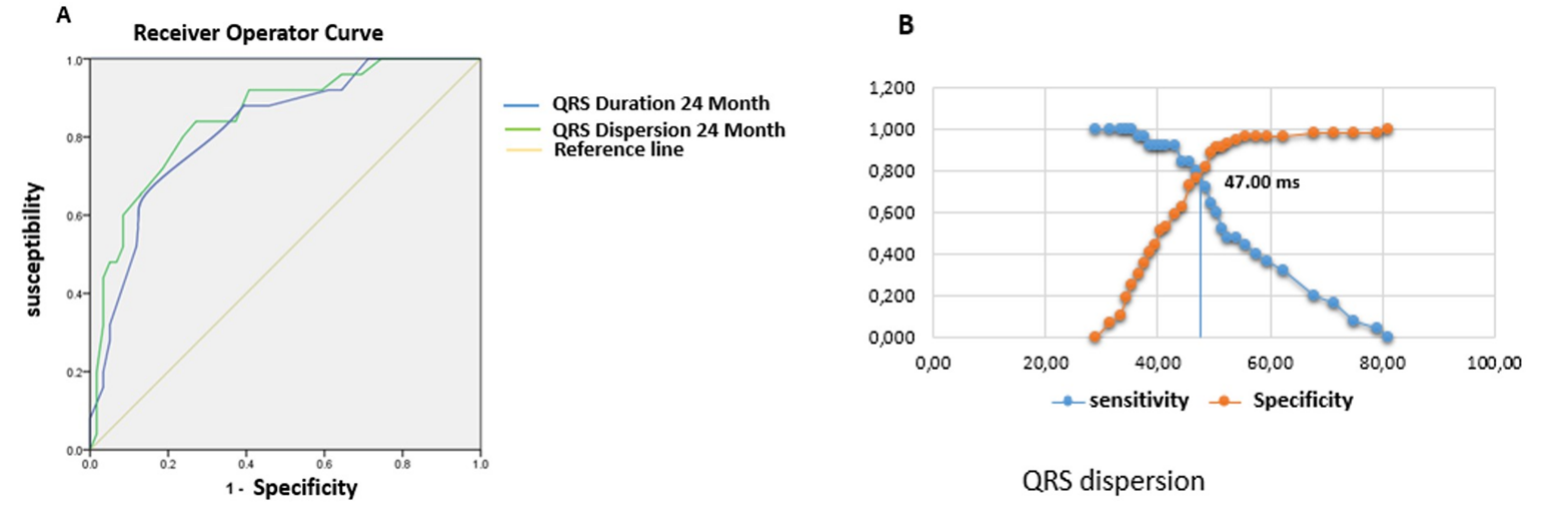
Receiver Operating Characteristic of QRS duration and dispersion for LVEF as a binary variable and cut-off point of QRS dispersion to predict LVEF worsening **A. **ROC curve of the discrimination power of QRS duration and dispersion for LVEF;** B. **QRS dispersion cut-off point to predict LVEF worsening

The ROC curve (Figure 3A) shows the discrimination power for QRS duration and dispersion, considering left ventricular ejection fraction as a dichotomous variable (left ventricular ejection fraction < 40% or ≥ 40%). The area under the curve values are shown in the Table 4.

**Figure 4:**
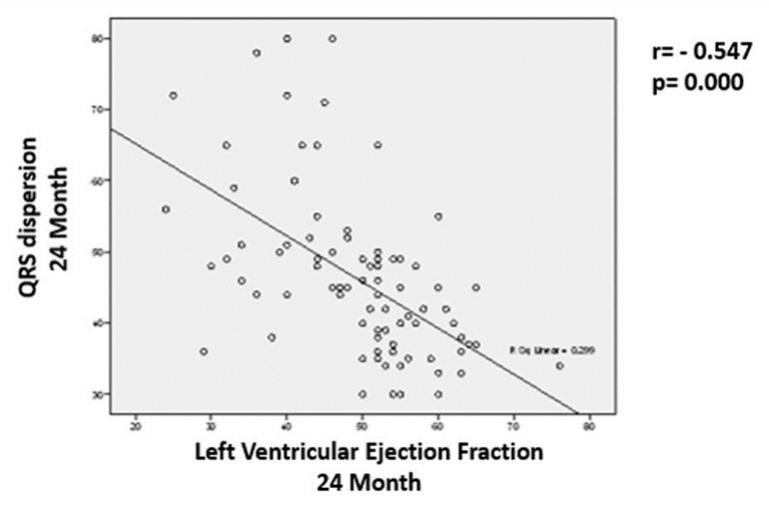
Linear regression for QRS dispersion and LVEF at 24 months

Figure 3B shows the QRS dispersion cut-off point to predict left ventricular ejection fraction worsening in the sample studied (47 milliseconds). Figure 4 shows the linear regression for QRS dispersion and left ventricular ejection fraction at 24 months of follow-up in the studied sample (r= -0.547; p=0.000). The formula for predicting left ventricular ejection fraction is: Left ventricular ejection fraction = - 0.950 * QRS dispersion + 83.936.

## DISCUSSION

In this study we showed that in patients with permanent right ventricular apical pacing there is an increased mechanical dyssynchrony and a decrease in left ventricular ejection fraction. From the total number of patients studied, 15 of them (15.47%) presented a left ventricular ejection fraction less than 40% at 24 months of follow-up. A relationship was demonstrated between lower values of left ventricular ejection fraction and increased QRS duration and dispersion. In addition, inter- and intraventricular dyssynchrony observed during follow-up was related with increased values of QRS duration and dispersion. This is the first study reporting the relationship between QRS dispersion and left ventricular ejection fraction worsening in patients with permanent right ventricle apical pacing and establishing its potential value in assessing inter- and intraventricular electromechanical dyssynchrony.

Clinical effects of right ventricular apical pacing regarding precipitation of left ventricular dysfunction and heart failure are well-documented. A Multicenter Automatic Defibrillator Implantation Trial (MADIT)-II sub-study^16^ described the harmful effects of right ventricular apical pacing. During 20 months of follow-up, patients who had a higher cumulative rate of right ventricular apical pacing compared with patients who had infrequent pacing, suffered a higher incidence of heart failure, ventricular arrhythmias and mortality.

**Table 3:** Correlation among some variables, at the beginning and the end of the study

y (dependent)	x (independent)	t	p	Pearson coefficient
**Positive correlation**				
QRS dispersion	Interventricular dyssynchrony	2,496	0,015	0,454
QRS dispersion	Intraventricular dyssynchrony	3,301	0,001	0,446
QRS dispersion	Frequency of pacing	3,013	0,003	0,467
				
**Negative correlation**				
Left Ventricular Ejection Fraction	Frequency of pacing	12,605	0,000	-0,589
Left Ventricular Ejection Fraction	QRS dispersion	-2,451	0,016	-0,606

**Table 4:** Discriminatory power of QRS duration and QRS dispersion, estimated using the receiver operator curve (ROC) using estimates and the 95% confidence interval (CI) and area under the curve

Variables	Area under the Curve	Typical Error	Significance	CI 95% Inferior Limit	CI 95% Superior Limit
24-Month QRS Duration	0.790	0.051	0.000	0.740	0.935
24-Month QRS Dispersion	0.850	0.046	0.000	0.761	0.940

The deleterious effects of chronic right ventricular apical pacing were also reflected in the study by Chen S et al.^17^, which reported an increase of the prevalence of heart failure or left ventricular dysfunction in permanent pacing, corelated with pacing percentage. PACE study (Pace to Avoid Cardiac Enlargement)^18^, a multicenter, double-blind, randomized trial was designed to determine if biventricular pacing was superior to permanent right ventricular apical pacing for preventing structural changes of left ventricular and to avoid systolic disfunction. Patients with left ventricular ejection fraction ≥45%, with pacemaker indication due to high-grade of AV block or sinus node dysfunction were included. All patients received atrial and biventricular pacing lead, and the pacemaker could be programmed for RV or biventricular pacing. Two days after device implanting, patients were stratified according to whether diastolic dysfunction was present. In each group the patients were randomized to receive right ventricular apical pacing or biventricular pacing (1:1). At 12 months, the group with right ventricular apical pacing (n = 88) versus biventricular pacing (n = 89) had lower left ventricular ejection fraction (54.8 ± 9.1% versus 62.2 ± 7%, p < 0.001); 8 patients who received right ventricular apical pacing (9%) versus 1 patient who underwent biventricular pacing (1%) had a left ventricular ejection fraction less than 45%. The deleterious effects of right ventricular apical pacing occurred in both pre-specified groups, including patients with and without previous diastolic dysfunction.

Using QRS duration for estimating the degree of dyssynchrony induced by pacing is not a new concept. Lange and al.^19^ found a QRS duration of 156 ± 22 ms in the group who underwent permanent right ventricular apical pacing, higher than in our group, perhaps due to a reduced sample of patients, only 9 of them having apical pacing. In addition, his patients being older, QRS duration could have been increased because of age-related changes of heart electrical system conduction and the cardiac architecture. Molina Mora et al.^20^ obtained an average QRS of 135.5 ± 26.2 ms, similar to our results, although in their group QRS duration did not significantly increased (135.5 up to 141.8 ms, p = 0.4), and there were no significant changes in the left ventricular ejection fraction (62.4 versus 59.2%, p = 0.17).

QRS dispersion is a simple electrocardiographic marker with potential value in the assessment of patients in different clinical settings^21^. Right ventricular apical pacing causes the heart depolarization to be reversed (depolarization proceeds from the right ventricular apical up to the base of the heart). Changes in depolarization sequence will delay left ventricular depolarization on the upper septum part and the lateral regions and could explain the increased QRS duration and dispersion^22^. The depolarization changes the impact on the heart contraction mechanics and at the same time on the flow patterns, leading to left ventricular ejection fraction worsening^23^. Chavez et al.^14^studied 24 patients with depressed left ventricular ejection fraction secondary to right ventricular apical pacing. They demonstrated a higher QRS duration (178.12 ± 14.31 ms versus 103.17 ± 3.43 ms) and QRS dispersion (45.07 ± 4.83 ms versus 33.15 ± 2.14 ms) during right ventricular apical pacing compared to septal region pacing. Moreover, there was reverse remodeling and an increase in left ventricular ejection fraction with septal pacing.

Considering the results of the present study, we consider that depolarization delay in right ventricular apical pacing is represented by higher values of QRS dispersion and that this could identify electromechanical dyssynchrony. The ROC curve shown in Figure 3A for QRS duration at 24 months of follow-up, took into account the wider QRS value on 12 the lead-ECG. Thus, the area under the curve for QRS duration depends on a single measured value. However, QRS dispersion depends on the difference between the QRS maximum and minimum values measured on the 12-lead ECG. Observing the greater discrimination power of the QRS dispersion in the ROC curve, we consider that we should not only measure the maximum value of the QRS duration, but also its dispersion. In the present study, we established a QRS dispersion > 47 ms as a predictor of left ventricular dysfunction and long-term electromechanical dyssynchrony in patients with right ventricular apical pacing (sensitivity 80% and specificity 76%).

## LIMITATIONS OF THE STUDY

Our study was performed with a relatively small sample size and with a short time of follow-up, therefore the results obtained need to be reproduced and evaluated in a larger number of patients with different characteristics and comorbidities.

## CONCLUSION

An elevated percentage of right ventricular apical pacing increased QRS duration and dispersion, both being related with echocardiographic mechanical dyssynchrony and left ventricular ejection fraction decrease. This study is important because it shows that QRS dispersion could be a better predictive variable than QRS duration for identifying left ventricular ejection fraction worsening in patients with permanent right ventricular apical pacing.

## Key Points

**◊** *Permanent right ventricular apical pacing produces an unphysiological ventricular activation sequence which could lead to systolic dysfunction and precipitate heart failure*

**◊** *Right ventricular apical pacing increases QRS duration and dispersion, both being related to mechanical dyssynchrony and left ventricular ejection fraction decrease*

**◊** *QRS dispersion was better than QRS duration for identufying mechanical dyssynchrony and ejection fraction worsening*

**◊** *Studies should be designed to evaluate the same electrocardiographic variables investigated here, in order to establish pacing sites that maintain electromechanical synchrony. The pacing site could be the His bundle which has been studied in several worldwide implantation centers*
